# The Saline‐Immersion/Irrigation TEchnique for Endoscopic Submucosal Dissection of Colorectal Lesions: Outcomes From a Large Western Cohort

**DOI:** 10.1111/jgh.70163

**Published:** 2025-12-03

**Authors:** Elisabet Maristany Bosch, Georgios Kalopitas, Alessandro Rimondi, Hironori Yamamoto, Alberto Murino, Edward J. Despott

**Affiliations:** ^1^ Royal Free Unit for Endoscopy The Royal Free Hospital and University College London (UCL) Institute for Liver and Digestive Health London UK; ^2^ Division of Gastroenterology, Department of Medicine Jichi Medical University Shimotsuke Japan; ^3^ Digestive Disease and Surgery Institute Cleveland Clinic London UK

**Keywords:** colorectal ESD, conscious sedation, endoscopic submucosal dissection, pocket‐creation method, saline immersion, SITE, SITE‐PCM

## Abstract

**Background and Study Aims:**

Endoscopic submucosal dissection (ESD) is increasingly used for the resection of complex lesions of the gastrointestinal tract. However, its adoption in the management of colonic lesions in Western practice remains challenging, mainly due to the anatomical complexities of the colon. The saline‐immersion/irrigation technique (SITE), combined with the pocket‐creation method (PCM) may help to overcome these challenges. This study evaluates outcomes of SITE‐PCM ESD in a Western tertiary center.

**Methods:**

We analyzed consecutive colorectal ESD procedures performed at our center between 2017 and 2024. SITE‐PCM was used in all cases. Lesion characteristics, procedural outcomes, histopathological findings, adverse events, and follow‐up data were analyzed.

**Results:**

A total of 181 lesions in 177 patients were resected en bloc using SITE‐PCM ESD. Sixty lesions (33.1%) were located in the proximal colon, while 79 (43.6%) were rectal lesions. R0 and curative resection rates were 92.3% and 90.6%, respectively. The median procedure time was 120 min. Adverse events occurred in 4.4% of cases, with only three patients necessitating hospitalization and nonsurgical intervention due to delayed bleeding, and one patient requiring surgical management after a delayed perforation. Notably, 93.4% of the procedures were performed under conscious sedation.

**Conclusions:**

In our experience SITE‐PCM‐ESD appears to be a safe and effective minimally invasive approach for complex colorectal lesions. It achieves high R0 and curative resection rates with low adverse events and is feasible under conscious sedation. These findings support broader adoption of SITE‐PCM in Western practice and justify prospective multicenter validation.

**Abbreviations**
ASA: American Society of Anesthesiologists
CO_2_
: carbon dioxide
CS: conscious sedation
ESD: endoscopic submucosal dissection
GA: general anesthesia
HGD: high‐grade dysplasia
IQR: interquartile range
LGD: low‐grade dysplasia
LST‐G: granular laterally spreading tumors
LST‐NG: non‐granular laterally spreading tumors
NETs: neuroendocrine tumors
PCM: pocket‐creation method
SD: standard deviation
SITE: saline‐immersion/irrigation technique
SSL: sessile serrated lesion
TAMS: trans‐anal microsurgery
U‐EMR: underwater endoscopic mucosal resection
WHO: World Health Organization

## 1 Introduction

Endoscopic submucosal dissection (ESD) has become a well‐established technique for en bloc, curative resection of esophageal, gastric, colonic, and rectal lesions [1, 2]. This approach enables en bloc resection with high R0 resection rates, low recurrence rates, and consequently, increased surveillance intervals. While ESD is widely adopted for these indications, its application in the colon remains challenging, particularly in Western countries. This is mainly due to the anatomical complexities of the colon, the thin muscularis propria, the presence of angulations, and the limitations in scope maneuverability, which pose significant technical difficulties, impacting both lesion access and procedural stability, with longer procedural times and higher adverse events.

Various techniques have been developed to overcome these challenges to ESD. The pocket‐creation method (PCM), introduced by Yamamoto's group in 2014 [3, 4], facilitates ESD by preserving the submucosal lift, enabling intrinsic traction, reducing respiratory interference, and improving procedural efficiency. Furthermore, immersion techniques have also evolved to address drawbacks of gaseous insufflation in conventional ESD. Underwater endoscopic mucosal resection (U‐EMR), introduced by Binmoeller [5], and subsequent underwater ESD adaptations for esophageal and rectal lesions represented such innovations [6, 7, 8, 9].

**FIGURE 1:**
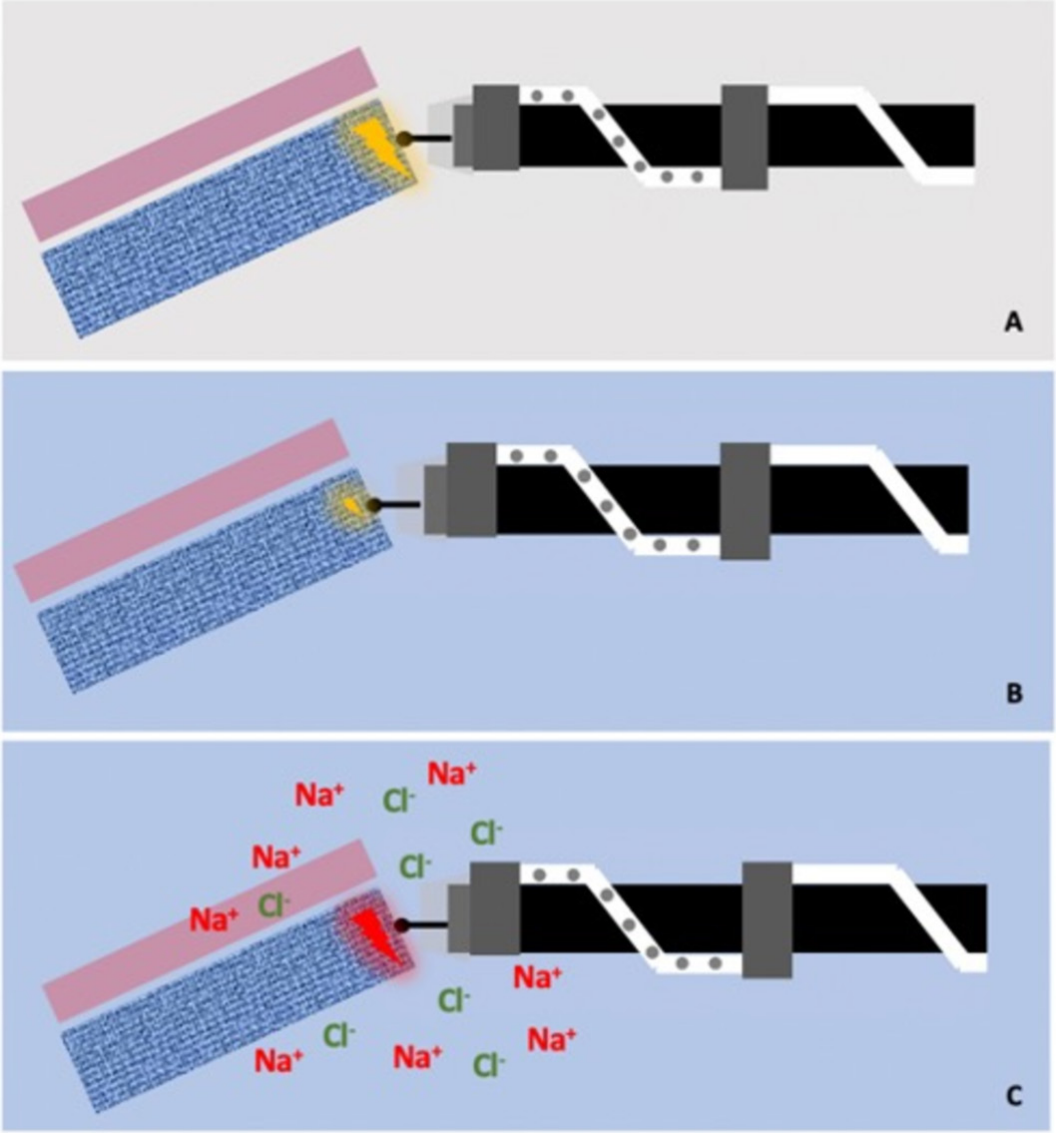
(A) Conventional ESD with CO_2_—conduction of diathermy is provided by the tissue‐surface electrolytes. (B) ESD underwater—in underwater ESD, the diathermic current does not transmit as surface electrolytes are diluted away from the tissue surface. (C) Saline‐immersion/irrigation (SITE)‐ESD—saline solution provides an electrolyte‐rich environment, which enhances diathermic conductivity and knife function.

While deionized water is suitable for U‐EMR, its lack of electrical conductivity renders it suboptimal for ESD, necessitating the use of high‐power diathermy settings. To address this limitation, we introduced the saline‐immersion/irrigation technique (SITE) in 2017 [10], replacing carbon dioxide (CO_2_) and water with sterile isotonic saline (0.9%) as a conductive medium for ESD (Figure 1). The term “saline” in the acronym of the name highlights its central role in enabling effective electrosurgical dissection.

In addition to enhanced conductivity, when combined with the PCM, SITE confers several additional advantages: It improves visualization by eliminating the gas–liquid interface and smoke interference, and it facilitates natural traction through flap buoyancy and increases patient comfort. This latter benefit arises because CO₂, a highly expansile gas, generates higher intraluminal pressure than saline—an effect explained by the ideal gas law (PV = nRT) [11].

Since 2017, we have adopted SITE‐PCM ESD as our standard approach for all colorectal ESDs. This study presents our single‐center cohort data on SITE‐PCM ESD for colorectal lesions, evaluating its efficacy, safety profile, and its potential for broader application.

## 2 Materials and Methods

### 2.1 Methods

This single‐center cohort study included consecutive patients referred to our tertiary unit for ESD of colorectal lesions ≥ 2 cm or histologically confirmed rectal neuroendocrine tumors (NETs) between 2017 and 2024. Data were extracted from a prospective database. It should be noted that during the COVID‐19 pandemic, advanced endoscopic services were suspended at our unit for a 30‐month period (2020–2022), impacting procedural volumes. Lesions treated with EMR/hybrid techniques or involving the appendiceal orifice were excluded from our analysis. Demographic and clinical parameters were recorded. The Paris classification and lateral spreading tumor (LST) classification [12] were used to define the macroscopic appearance of lesions, and their location was categorized as follows: proximal colon (cecum, ascending, and transverse colon), distal colon (descending and sigmoid colon), and rectum. Patients' demographics including American Society of Anesthesiologists (ASA) [13] score, technical aspects including among others submucosal fibrosis grading [14], histopathological analysis, completeness of endoscopic resection, and procedure‐related adverse events were also recorded. All cases were firstly discussed at our advanced endoscopy multidisciplinary meeting (MDTM), where ESD was considered the most appropriate therapeutic approach, in accordance with ESGE guidelines [2].

### 2.2 Colorectal ESD Procedures

**FIGURE 2:**
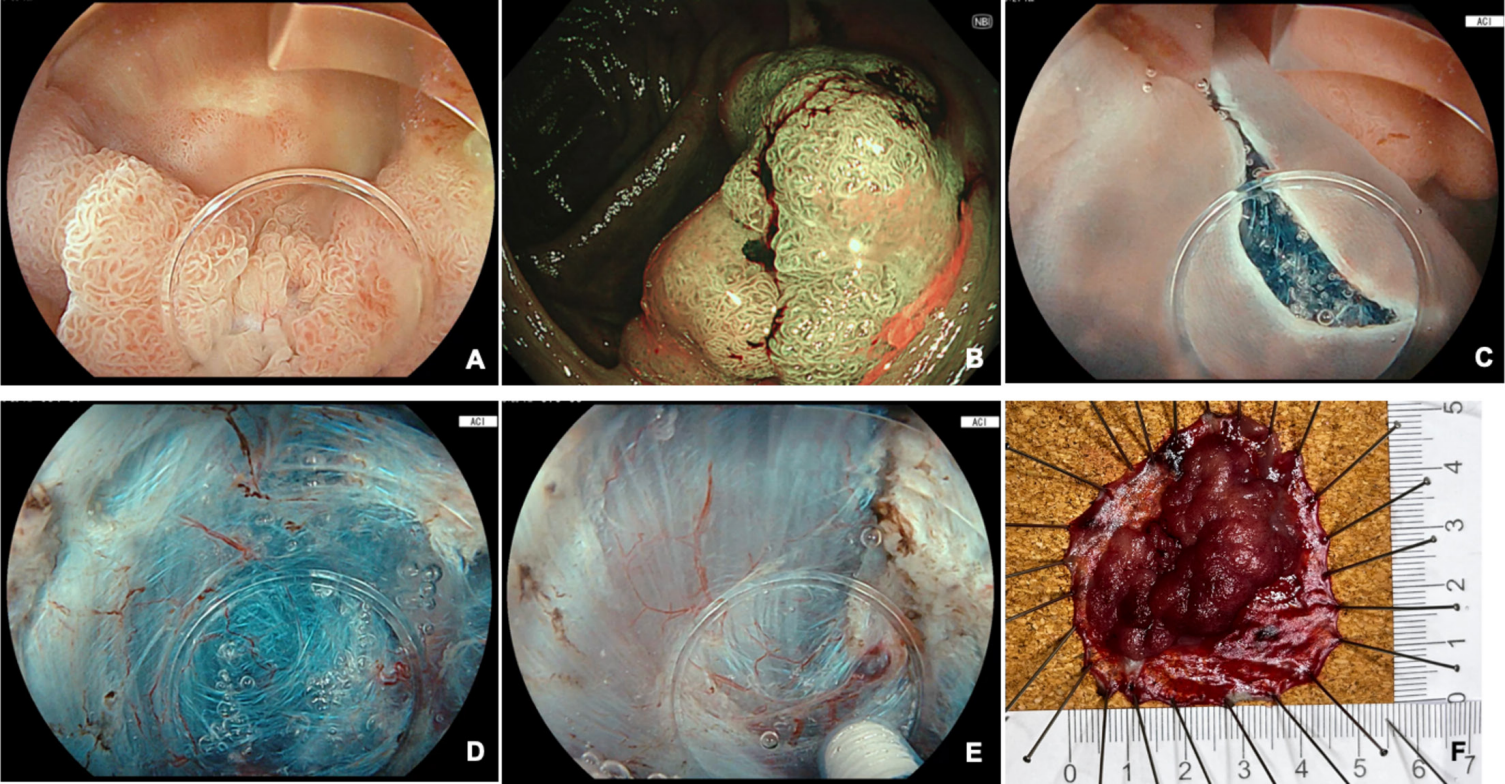
SITE‐PCM‐ESD: (A) lesion assessment, LST‐G‐M (Paris 0‐IIa‐Is) in the ascending colon; (B) JNET 2A on Narrow Band Imaging (NBI); (C) mucosal incision on the anal side using amber‐red color imaging (ACI) light; (D) pocket‐creation method (PCM), the submucosal space within the pocket as seen with ACI; (E) knife‐tip precoagulation of submucosal vessels as seen with ACI; (F) resected specimen pinned to cork.

**FIGURE 3:**
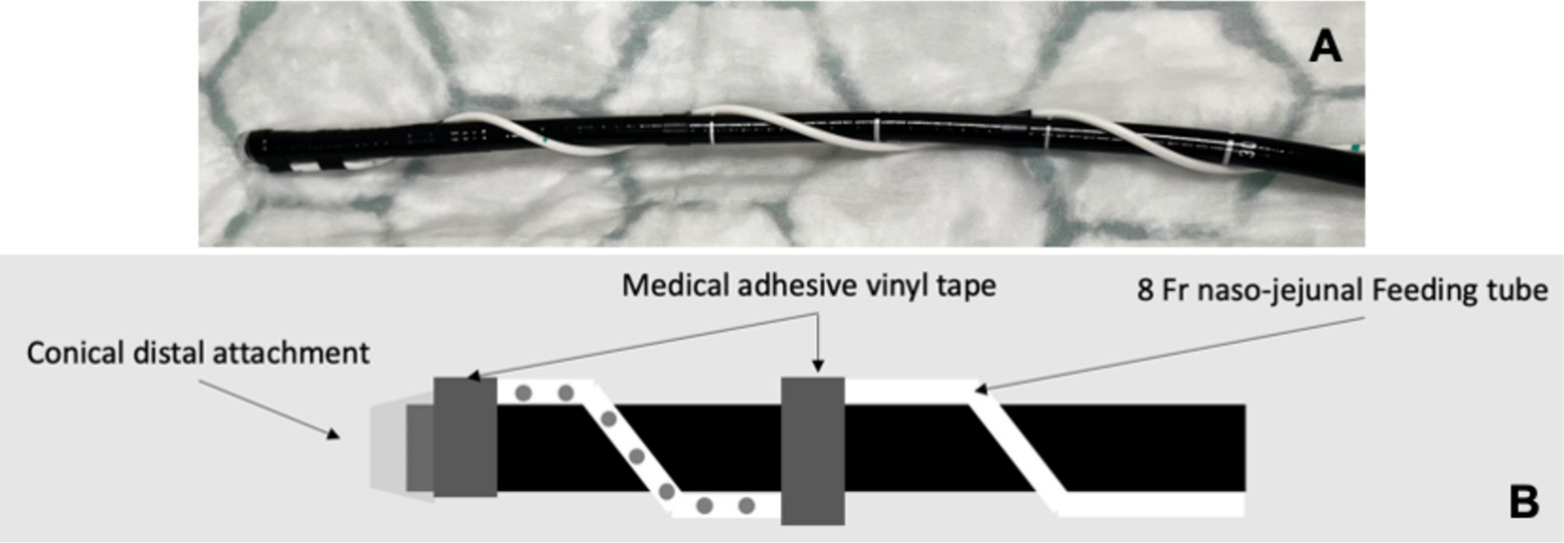
(A) Picture and (B) graphic representation of the “Asclepius tube” [16]: A nasojejunal drainage tube wrapped around the endoscope and fixed with vinyl tape. A conical distal attachment has been previously placed and secured with vinyl tape.

ESD was performed by two expert endoscopists (EJD, AM) under operator‐delivered conscious sedation (CS) or general anesthesia (GA). Single‐channel endoscopes with a dedicated saline‐jet were used (Fujifilm Healthcare, Tokyo, Japan or Olympus Medical, Tokyo, Japan). The endoscope was equipped with a conical short, transparent hood (ST‐hood, Fujifilm Healthcare, Tokyo, Japan) to facilitate ESD using the PCM [3]. SITE‐PCM was employed as standard practice in our center for all colorectal ESDs, replacing intraluminal gas with continuous irrigation of isotonic saline solution (0.9% sodium chloride), completely flooding the relevant part of the colorectal lumen (Figure 2). From 2023, drainage tubes were introduced to prevent bowel overdistension [15]. Our current standard is the “Asclepius tube,” an 8fr fenestrated nasojejunal tube wrapped around the scope, secured with vinyl tape, and connected to a low‐flow suction (Figure 3) [16]. Pre‐filled syringes of submucosal lifting agent (EverLift, Laborie, Portsmouth, USA) were used for initial submucosal injection, and a solution of 4% succinylated gelatin, 1:200000 adrenaline, and indigo carmine dye was employed as lifting solution for injection through the knife. Needle‐type knives: 1.5 mm (ball‐tipped FlushKnife BTs 1.5 mm, Fujifilm Healthcare, Tokyo, Japan) or the I‐type HybridKnife Flex, (ERBE Elektromedizin, Tübingen, Germany) were used. For mucosal incision and nonvascular dissection diathermic settings were tailored for more precise cutting with minimal coagulation (EndoCut I effect 1, duration 4, interval 1, VIO 3, ERBE Elektromedizin, Tübingen, Germany) and for precoagulation and vessel management we used knife‐tip/side coagulation using SwiftCoag effect 2.5 to 3.5 (VIO3, ERBE Elektromedizin, Tübingen, Germany) combined with continuous saline irrigation, to achieve an optimal coagulation effect. A representative video demonstrating our technique is provided (Video 1).

**VIDEO 1:** Representative video demonstrating the saline‐immersion/irrigation technique (SITE) for ESD of a large lesion of the ascending colon. This video demonstrates key steps of SITE‐PCM, including set up of the endoscope with drainage tube, assessment, submucosal injection, initial incision, pocket‐creation, dissection, and complete resection of a colonic lesion under conscious sedation.

Once ESD was completed, the resection site was carefully examined and any vessel‐coagulation applied if deemed necessary. Full or partial resection‐site closure with through‐the‐scope clips (Resolution, Boston Scientific, MA, USA; LOCKADO, Micro‐Tech, Nanjing, China) was selectively performed and hemostatic peptide‐matrix gel (PuraStat, 3D‐Matrix Ltd., Tokyo, Japan) applied, to potentially promote healing and mitigate the risk of delayed adverse events [17, 18], when the resection site was not closed with clips. Specimens were retrieved using a dedicated net (RothNet, Steris, Dublin, Ireland) and were measured after being pinned onto cork. Lesion area was calculated using the ellipse formula A = abπ ((maximum diameter/2)*(minimum diameter/2)* π). The resection time was measured from the initial incision to complete lesion resection. The resection speed was then calculated by dividing the lesion area (in mm^2^) by the total resection time in minutes (mm^2^/min).

### 2.3 Histopathological Study

The pinned specimen was placed in 10% formalin for processing, analysis, and reporting by expert gastrointestinal histopathologists, according to the WHO 2019 classification system for the digestive system tumors [19] and the Royal College of Pathologist Dataset for histopathological reporting of colorectal cancer. Macroscopic features and size were recorded. Lateral and vertical margins were evaluated. Resection was defined as R0 with lesion‐free 1000‐μm vertical and lateral margins, R1 with evidence of lesion cells on the vertical or lateral margins, and additionally curative resection was reported if the specimen met the R0 definition with no other risk factors for lymph node metastasis: no lymphovascular/perineural invasion, well differentiation, absence of budding, and no submucosal infiltration deeper than 1000 μm. The presence of the abovementioned risk factors for lymph node metastasis or submucosal cancer with or without positive vertical margins was defined as a high‐risk histopathological finding.

### 2.4 Adverse Events

Adverse events (defined by the AGREE classification) [20] were categorized as either immediate (detected during the procedure) or delayed (detected later). Severe intraprocedural bleeding was defined as endoscopically untreatable bleeding occurring during ESD, leading to procedure interruption, blood transfusion, or hospital admission. Delayed bleeding was defined as clinical signs of bleeding, such as hematochezia and a hemoglobin drop of more than 20 g/L, requiring further endoscopic, radiological, or surgical intervention within 2 weeks after ESD [21, 22]. Intraprocedural perforation was defined as endoscopic evidence of a transmural breach during ESD (Sydney 4) [23]. Delayed perforation was characterized by radiologically confirmed perforation accompanied by severe abdominal pain/peritonitis within 72‐h post‐ESD [24]. Other adverse events deemed to be related to the procedure were also recorded.

### 2.5 Hospitalization

Hospital admission was based on procedure outcome/need for clinical observation, as assessed by the endoscopist. According to our protocol, admission was prearranged beforehand for patients who lived more than 30 min away from the hospital and in specific cases, such as for elderly or comorbid patients. Post‐ESD, all patients received a standard 5‐day antimicrobial course of broad‐spectrum antibiotics targeting both aerobic and anaerobic flora as per our unit's protocol. Those with an uneventful procedure and stable clinical status were managed as day cases.

### 2.6 Post‐ESD Follow‐Up and Recurrence

Follow‐up was guided by histopathological findings as reviewed at our advanced endoscopy MTDM and in accordance with ESGE guidelines and AGA guidelines with the first endoscopic assessment planned between 6 and 12 months depending on histopathological findings [2, 25].

### 2.7 Statistics and Ethics

All data analysis was conducted with IBM SPSS Statistics version 30.0 (SPSS Inc., Chicago, Illinois, USA). Continuous variables were reported as means ± standard deviations (SD) for normally distributed data or medians ± interquartile ranges (IQR) for nonnormally distributed data. Categorical variables were expressed as frequencies and percentages.

Preprocedure, all patients gave their full, written informed consent, and this study was conducted following the principles of the latest version of the Declaration of Helsinki [26]. All data were coded, and patient anonymity was guaranteed.

## 3 Results

### 3.1 Patient and Lesion Characteristics

**FIGURE 4:**
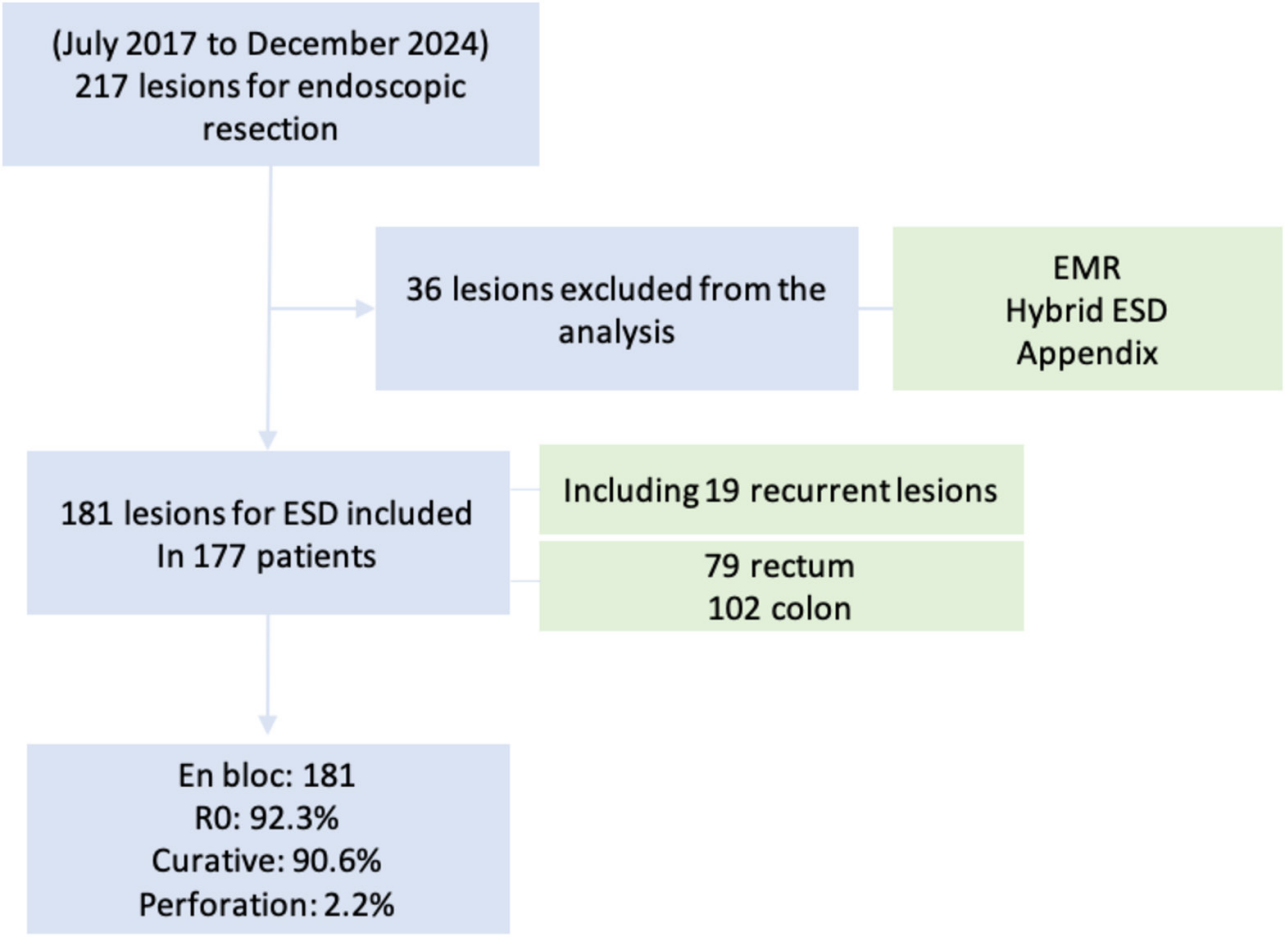
Flowchart of the study.

Between 2017 and 2024, 217 lesions were referred for advanced endoscopic resection. Thirty‐six lesions were excluded from our analysis, since these were resected using EMR variants/hybrid techniques after being deemed unsuitable for ESD (due to patient/lesion‐related factors) or involved the appendiceal orifice. Ultimately, 181 lesions in 177 patients were included in our analysis (Figure 4).

Nineteen (10.5%) lesions were recurrent: 13 post‐EMR (7.2%) and six post‐trans‐anal microsurgery (TAMS) (3.3%). The mean patient age was 65.2 years (SD 13.5, range 21–88), with 43.6% women (*n* = 79) and 56.4% men (*n* = 102). ASA‐2 score was the most prevalent in our analysis (50.3%). Seventy‐nine lesions (43.6%) were resected from the rectum, and 102 lesions were resected from the colon (56.4%), including proximal (*n* = 60, 33.1%) and distal (*n* = 42, 23.2%) colon. The median maximum lesion diameter was 40 mm (IQR 30–55, range 3–145 mm), with a median lesion area of 962 mm^2^ (IQR 471–1885, range 7–8954 mm^2^). Mucosal lesion morphology included 26 Paris 0‐Is (14.4%), 95 granular laterally spreading tumors (LST‐G) (52.5%), 34 non‐granular laterally spreading tumors (LST‐NG) (18.8%), and 10 Paris 0‐Isp lesions (5.5%). Sixteen (8.8%) lesions were NETs. Patient and lesion characteristics are detailed in Table 1.

**TABLE 1:** Patient and lesion characteristics.

Data	*N* (%)	
Total number of lesions referred	217	Excluded 36 (EMR/hybrid/appendix)
Total number of lesions included	181	
Total number of patients	177	
Recurrent	19 (10.5%)	
Gender		
Men	102 (56.4%)	
Women	79 (43.6%)	
Age (mean, years)	65.2 (SD 13.5, range 21–88)	
ASA score (median)	1: 54/181 (29.8%)	
	2: 91/181 (50.3%)	
	3: 7/181 (3.9%)	
	Not recorded: 29 (16%)	
Tumor location		
Rectum	79 (43.6%)	
Colon	102 (56.4%)	
Proximal colon	60 (33.1%)	Caecum 23 (12.7%), ascending colon 25 (13.8%), transverse colon 12 (6.6%)
Distal colon	42 (23.2%)	Descending colon 15 (8.3%), sigmoid colon 27 (14.9%)
Macroscopic type		
Sessile	26 (14.4%)	
LST‐G‐H	45 (24.8%)	
LSTG‐M	50 (27.6%)	
Total LST‐G	95 (52.5%)	
LST‐NG‐FE	19 (10.5%)	
LST‐NG‐PD	15 (8.3%)	
Total LST‐NG	34 (18.8%)	
Semipedunculated	10 (5.5%)	
Submucosal	16 (8.8%)	
Paris classification		
0‐IIa	43 (23.8%)	
0‐IIa‐IIb	2 (1.1%)	
0‐IIa‐IIc	26 (14.4%)	
0‐IIa‐Is	35 (19.3%)	
0‐IIb	12 (6.6%)	
0‐IIb‐IIc	3 (1.7%)	
0‐Is	34 (18.8%)	
0‐Isp	10 (5.5%)	
JNET classification		
2A	85 (47%)	
2B	38 (21%)	
Tumor max. diameter (median, mm)	40 (IQR 30–55, range 3–145)	
Area (median, mm ^2^ )	962 (IQR 471–1885 range 1–8954)	

Abbreviations: FE, flat‐elevated; H, homogeneous; IQR, interquartile range; LST‐G, granular laterally spreading tumors; LST‐NG, non‐granular laterally spreading tumors; M, mixed nodular; PD, pseudo‐depressed; SD, standard deviation.

### 3.2 ESD Outcomes

A total of 181 lesions were removed en bloc with ESD. R0 resection and curative resection rates were achieved in 167 (92.3%) and 164 (90.6%), respectively. R0 resection by lesion location was as follows: rectum: 72/79 (91.2%); colon: 95/102 (93.1%). Median procedural time was 120 min (IQR 75–180, range 20–590), and the median resection speed was 9 mm^2^/min (IQR 4.9–13.6, range 0.1–36.65). Severe fibrosis F2 was noted in 49 (27.1%) cases. Post‐ESD defect closure was performed in 106 cases (58.6%), complete closure in 56 (30.9%), and partial closure in 50 (27.7%). Peptide‐matrix gel was applied selectively postprocedure in 80 (44.2%). The characteristics of resected colorectal lesions, outcomes, and procedural parameters are described in detail in Table 2.

**TABLE 2:** ESD outcomes.

Data	*N* (%)	
R0 resection	167 (92.3%)	
R0 rectum	72/79 (91.2%)	
R0 colon	95/102 (93.1%)	
Proximal colon	55/60 (91.6%)	Caecum 21/23 (91.3%), ascending colon 23/25 (92%), transverse colon 11/12 (91.6%)
Distal colon	40/42 (95.2%)	Descending colon 15/15 (100%), sigmoid colon 25/27 (92.5%)
R1 resection	14 (7.7%)	
Vertical R1	4 (28.5%)	
Curative resection	164 (90.6%)	
Resection time (median, minutes)	120 (IQR 75–180, range 20–590)	
Resection speed (median, mm^2^/min)	9 (IQR 4.9–13.6 range 0.05–36.65)	
Submucosal fibrosis		
F0 (none)	108 (59.7%)	
F1 (mild)	24 (13.3%)	
F2 (severe)	49 (27.1%)	
Clip closure	106 (58.6%)	
Complete	56 (30.9%)	
Partial	50 (27.7%)	
Histopathological findings		
LGD	124 (68.5%)	
HGD	24 (13.3%)	
SSL	5 (2.8%)	
SSL + dysplasia	3 (1.7%)	
NET	16 (8.8%)	
Slightly invasive (< 1000 μm) submucosal carcinoma	2 (1.1%)	
Deeply invasive (≥ 1000 μm) submucosal carcinoma	7 (3.9%)	
Adverse events	8 (4.4%)	
Delayed bleeding	3 (1.7%)	
Intraprocedural perforation	3 (1.7%)	
Delayed perforation	1 (0.6%)	
Other	1 (0.6%)	
Hospital admission	65 (35.9%)	
Days of stay (median, days)	2 (IQR 1–2, range 1–20)	
Anesthetics		
GA	12 (6.6%)	
Conscious sedation	169 (93.4%)	
Midazolam (median, mg)	6 (IQR 4–8.5, range 1–12.75)	
Fentanyl (median, mcg)	150 (IQR 100–200, range 25–400)	
Elective surgery	5 (2.7%)	
R1	3/14 (21.4%)	
R0 noncurative	2/167 (1.2%)	

Abbreviations: GA, general anesthesia; HGD, high‐grade dysplasia; LGD, low‐grade dysplasia; NETs, neuroendocrine tumor; SSL, sessile serrated lesion.

### 3.3 Adverse Events and Hospitalization

Adverse events occurred in eight cases (4.4%): Three (3.3%) intraprocedural perforations (Sydney 4), which were all managed endoscopically with through‐the‐scope clip closure, routine prophylactic antibiotics, and observation without further consequences; three patients (1.7%) experienced delayed bleeding, requiring hospitalization and nonsurgical intervention. Peptide‐matrix gel had been applied in two of these cases, although no meaningful association regarding delayed bleeding could be established in our cohort. One (0.6%) patient suffered a delayed perforation of the sigmoid colon following the use of coagulation forceps—this warranted laparotomy with a Hartmann's procedure. One patient with associated comorbidities was hospitalized for 20 days due to decompensation of underlying cardiac failure. Sixty‐five of 181 patients (35.9%) were admitted postprocedure. The median length of stay was 2 days (IQR 1–2, range 1–20 days).

### 3.4 Sedation

A total of 169 (93.4%) procedures were performed under operator‐delivered CS, using a combination of fentanyl and midazolam. The median fentanyl dose was 150 mcg (IQR 100–200, range 25–400), and the median dose of midazolam was 6 mg (IQR 3.75–8.5, range 1–12). No procedures performed under CS had to be interrupted/stopped due to patient discomfort. Twelve procedures (6.6%) were performed under GA.

### 3.5 Histopathology Results

A total of nine (5%) patients presented high‐risk histopathological findings. Two (1.1%) lesions had features of superficially invasive (< 1000 μm) submucosal carcinoma. In the first patient, we achieved R0‐curative resection, while in the second patient, despite R0 resection, lymphovascular invasion was present and required surgery. Seven (3.9%) lesions were deeply invasive (≥ 1000 μm) submucosal carcinomas. Sixteen (8.8%) of the resected lesions were NETs. The rest of the histopathological findings are detailed in Table 2.

### 3.6 Elective Surgery

Five (2.7%) patients underwent elective surgery postprocedure due to unfavorable histopathological findings. Three additional patients with high‐risk features did not undergo surgery, as they were deemed unfit for operative management and were instead managed conservatively, with no recurrence in any of them after a median follow‐up of more than 24 months.

### 3.7 Follow‐Up

A total of 104 of 177 patients (58.8%) have undertaken their planned endoscopic follow‐up to date. The median follow‐up time was 10 months (IQR 5–14 months). Among the 14 patients with R1 resections, three underwent curative elective surgery (patients with deeply invasive submucosal carcinomas and positive vertical resection margins). The remaining 11 have had endoscopic follow‐up, with only one of them experiencing a local recurrence; this was successfully treated with EMR. It should be noted that this lesion involved the ileocecal valve. No recurrence was found in any of the patients with R0 resections who had endoscopic follow‐up.

## 4 Discussion

Our large Western cohort included 181 colorectal lesions resected using SITE‐PCM ESD. In our experience, this achieved high rates of R0 (92.3%) and curative resection (90.6%), consistent with Japanese colorectal ESD series [27, 28, 29], and some recently published Western cohorts [30, 31, 32, 33, 34, 35]. Our lesions were notably large (median 40 mm) and predominantly colonic, with one‐third resected from the proximal colon. Interestingly, R0 resection rates were higher in the distal colon than in the rectum (95.2% vs. 92.3%), and notably 100% in the descending colon (15/15), indicating consistent technical success across different colorectal segments. Adverse events were rare. Only 1 patient (0.6%) required emergency surgery for delayed perforation, and 3 (1.7%) experienced delayed bleeding, requiring nonsurgical management. These outcomes also compare favorably with other Western series [36] and align closely with Japanese results [29].

The majority of procedures (92.8%) were performed under operator‐delivered conscious sedation (CS), offering an effective, less invasive alternative to GA. This approach was particularly beneficial in comorbid and frail patients (median ASA 2), allowing comfortable, safe treatment while conserving healthcare resources and facilitating quicker recovery, without compromising safety.

The efficacy, safety, and comfort observed in our cohort are likely attributable to the consistent use of SITE [10, 37]. In our experience, saline‐immersion/irrigation offers several procedural advantages. It significantly enhances visualization by eliminating smoke and gas–liquid interfaces typically generated during dissection in a gas‐filled lumen. Additionally, the differential refractive index of saline provides intrinsic image magnification, which can be further enhanced by using high‐definition endoscopes with optical zoom. The buoyancy of the mucosal flap in saline also creates natural traction, facilitating clearer access to the submucosal plane.

Furthermore, saline‐immersion/irrigation helps maintain a thick, electrolyte/liquid‐filled submucosal cushion and allows for lower intraluminal pressure as compared with gaseous insufflation. This creates a more stable working environment within the submucosal plane during dissection. The collapsed bowel, free from gaseous distension improves endoscope maneuverability allowing a more controlled approach to dissection—especially important in narrow or angulated colonic segments. Collectively, these features, in our experience contributed to safe and effective ESD, particularly in anatomically challenging regions.

In line with these technical benefits, we experienced a low perforation rate and no cases of severe intraprocedural bleeding requiring escalation of care. This may be explained by our saline‐assisted coagulation technique. Specifically, SITE enables highly effective precoagulation of vessels without frequent reliance on coagulation forceps. This is achieved by simultaneously irrigating saline through the endoscope's jet channel while concomitantly applying knife‐alone coagulation diathermy (SwiftCoag Effect 2.5–3.5, Erbe VIO3, ERBE Elektromedizin, Tübingen, Germany). This technique effectively seals vessels, producing a controlled whitening—which we refer to as the “Frozen Effect”—resembling a frost‐like spread along the vessel and its branches, visually confirming successful vessel precoagulation.

Our favorable outcomes support the value of SITE‐PCM in enabling en bloc resection of complex colorectal lesions by preserving organ integrity, while also minimizing postprocedural burden. Importantly, SITE also minimized recourse to GA and its associated risks. Our findings align with prior reports by Nagata et al. [38], Koyama et al., and others [28, 39, 40], which have demonstrated similarly low adverse event rates and strong oncological efficacy using SITE‐ESD technique.

While these results are encouraging and suggest that SITE is safe, effective, and comfortable for patients, our evaluation has several limitations. This study is a single‐center analysis of consecutive procedures performed at our tertiary‐referral unit and therefore may not fully reflect potential outcomes from lower volume centers with less‐experienced operators. However, our results remain consistent with previously published large series from other institutions, supporting their broader relevance and external validity [27, 28, 29, 30, 31, 32]. The relatively modest resection speed should in our opinion be viewed in the context of the lesion complexity of our cohort. Many lesions were referred due to prior failed resections or were in technically challenging regions like the proximal colon (33.1%). In such cases, prioritizing safety and complete resection over speed was a deliberate clinical choice. Finally, while follow‐up data are currently available for 58.8% of patients, this reflects the fact that approximately 40% of procedures were performed after the hiatus caused by the COVID‐19 pandemic, and surveillance is scheduled within the upcoming months. As follow‐up continues, more comprehensive long‐term outcomes will become available.

In summary, in our experience, SITE‐PCM ESD is highly effective, safe, comfortable, and resource‐efficient for minimally invasive, organ‐preserving en bloc endoscopic resection of complex colorectal lesions. From our clinical perspective, its favorable comfort profile obviates the need for GA, making it particularly suitable for broader clinical adoption in a Western setting. We therefore encourage multicenter, randomized controlled prospective studies to further evaluate the reproducibility and comparative performance of SITE‐PCM against conventional ESD, particularly in terms of patient comfort, overall outcomes, postprocedural recovery, and patient experience.

## 5 Conclusion

In conclusion, our data reflect that SITE‐ESD appears to be a safe and effective technique for en bloc resection of complex colorectal lesions, achieving high R0 and curative resection rates while maintaining GA‐free patient comfort and a low incidence of adverse events. Given this efficacy, safety, and patient‐centered profile, in our opinion, SITE‐ESD has the potential to become more widely adopted to provide optimal outcomes for colorectal ESD. Further prospective studies are warranted to validate and expand upon our findings.

## Funding

The authors received no specific funding for this work.

## Conflicts of Interest

The authors declare the following conflicts of interest:

E.M.B.: No disclosures.

G.K.: No disclosures.

A.R.: No disclosures.

H.Y.: Consultancy to Fujifilm Healthcare, from whom he has received honoraria, grants, and royalties.

A.M.: Personal payments/honoraria/fees: Olympus Medical, Laborie, Boston Scientific, Fujifilm Healthcare Europe.

E.J.D.: Educational grants in support of conference organization, and honoraria, from Fujifilm Healthcare Europe, Pentax, and Olympus Medical, and honoraria from Ambu and Fujifilm Healthcare Europe.

## Data Availability

The data that support the findings of this study are available from the corresponding author upon reasonable request.

## References

[1] P. Libânio , P. Pimentel‐Nunes , B. Bastiaansen , et al., “Endoscopic Submucosal Dissection Techniques and Technology: European Society of Gastrointestinal Endoscopy (ESGE) Technical Review,” Endoscopy 55, no. 4 (2023): 361–389, 10.1055/a-2031-0874. 36882090

[2] P. Pimentel‐Nunes , D. Libânio , B. A. J. Bastiaansen , et al., “Endoscopic Submucosal Dissection for Superficial Gastrointestinal Lesions: European Society of Gastrointestinal Endoscopy (ESGE) Guideline—Update 2022,” Endoscopy 54, no. 6 (2022): 591–622. 35523224 10.1055/a-1811-7025

[3] Y. Hayashi , K. Sunada , H. Takahashi , et al., “Pocket‐Creation Method of Endoscopic Submucosal Dissection to Achieve en Bloc Resection of Giant Colorectal Subpedunculated Neoplastic Lesions,” Endoscopy 46, no. Suppl 1 UCTN (2014): E421–E422. 25314173 10.1055/s-0034-1377438

[4] S. B. Javia , W. Reid , and W. Srikureja , “Saline‐Immersion Endoscopic Submucosal Dissection Using Pocket‐Creation Method,” VideoGIE 9, no. 1 (2024): 35–37. 38261865 10.1016/j.vgie.2023.09.008 PMC10794121

[5] K. F. Binmoeller , F. Weilert , J. Shah , Y. Bhat , and S. Kane , ““Underwater” EMR Without Submucosal Injection for Large Sessile Colorectal Polyps (With Video),” Gastrointestinal Endoscopy 75, no. 5 (2012): 1086–1091. 22365184 10.1016/j.gie.2011.12.022

[6] S. Yoshii , Y. Hayashi , T. Matsui , et al., ““Underwater” Endoscopic Submucosal Dissection: A Novel Technique for Complete Resection of a Rectal Neuroendocrine Tumor,” Endoscopy 48, no. Suppl 1 UCTN (2016): E67–E68. 26890547 10.1055/s-0042-101855

[7] T. Akasaka , Y. Takeuchi , N. Uedo , R. Ishihara , and H. Iishi , ““Underwater” Endoscopic Submucosal Dissection for Superficial Esophageal Neoplasms,” Gastrointestinal Endoscopy 85, no. 1 (2017): 251–252. 27449194 10.1016/j.gie.2016.07.018

[8] M. Kato , Y. Takatori , M. Sasaki , et al., “Water Pressure Method for Duodenal Endoscopic Submucosal Dissection (With Video),” Gastrointestinal Endoscopy 93, no. 4 (2021): 942–949. 32853646 10.1016/j.gie.2020.08.018

[9] N. Yahagi , T. Nishizawa , M. Sasaki , Y. Ochiai , and T. Uraoka , “Water Pressure Method for Duodenal Endoscopic Submucosal Dissection,” Endoscopy 49, no. 10 (2017): E227–E228. 28759932 10.1055/s-0043-113556

[10] E. J. Despott and A. Murino , “Saline‐Immersion Therapeutic Endoscopy (SITE): An Evolution of Underwater Endoscopic Lesion Resection,” Digestive and Liver Disease 49 (2017): 1376. 28967632 10.1016/j.dld.2017.08.035

[11] K. Garborg , M. F. Kaminski , W. Lindenburger , et al., “Water Exchange Versus Carbon Dioxide Insufflation in Unsedated Colonoscopy: A Multicenter Randomized Controlled Trial,” Endoscopy 47, no. 3 (2015): 192–199. 25412093 10.1055/s-0034-1390795

[12] S. Kudo , “Endoscopic Mucosal Resection of Flat and Depressed Types of Early Colorectal Cancer,” Endoscopy 25, no. 7 (1993): 455–461. 8261988 10.1055/s-2007-1010367

[13] D. S. Early , J. R. Lightdale , J. J. Vargo, 2nd , et al., “Guidelines for Sedation and Anesthesia in GI Endoscopy,” Gastrointestinal Endoscopy 87, no. 2 (2018): 327–337. 29306520 10.1016/j.gie.2017.07.018

[14] A. Matsumoto , S. Tanaka , S. Oba , et al., “Outcome of Endoscopic Submucosal Dissection for Colorectal Tumors Accompanied by Fibrosis,” Scandinavian Journal of Gastroenterology 45, no. 11 (2010): 1329–1337. 20626303 10.3109/00365521.2010.495416

[15] Y. Kagaya , Y. Hayashi , and T. Morikawa , “Trans‐Anal Foley Catheter Facilitates Endoscopic Submucosal Dissection of a Distal Rectal Tumor,” Digestive Endoscopy 35, no. 7 (2023): e155–e157. 37779453 10.1111/den.14680

[16] T. Morikawa , A. Murino , H. Ishii , et al., “The “Asclepius Tube” A Slim Drainage Tube Wrapped Around the Distal Part of the Endoscope for Cecal Endoscopic Submucosal Dissection,” VideoGIE 10, no. 2 (2025): 155–159, 10.1016/j.vgie.2024.10.002. 40012895 PMC11853374

[17] R. Maselli , L. Da Rio , M. Manno , et al., “Efficacy of Novel Endoscopic Hemostatic Agent for Bleeding Control and Prevention: Results From a Prospective, Multicenter National Registry,” Endoscopy International Open 12 (2024): C9. 39474488 10.1055/a-2406-7492 PMC11518632

[18] S. Subramaniam , K. Kandiah , F. Chedgy , et al., “A Novel Self‐Assembling Peptide for Hemostasis During Endoscopic Submucosal Dissection: A Randomized Controlled Trial,” Endoscopy 53, no. 1 (2021): 27–35. 32679602 10.1055/a-1198-0558

[19] I. D. Nagtegaal , R. D. Odze , D. Klimstra , et al., “The 2019 WHO Classification of Tumours of the Digestive System,” Histopathology 76, no. 2 (2020): 182–188. 31433515 10.1111/his.13975 PMC7003895

[20] K. J. Nass , L. W. Zwager , M. van der Vlugt , et al., “Novel Classification for Adverse Events in GI Endoscopy: The AGREE Classification,” Gastrointestinal Endoscopy 95, no. 6 (2022): 1078–1085.e8, 10.1016/j.gie.2021.11.038. 34890695

[21] M. X. Ma and M. J. Bourke , “Complications of Endoscopic Polypectomy, Endoscopic Mucosal Resection and Endoscopic Submucosal Dissection in the Colon,” Best Practice & Research Clinical Gastroenterology 30, no. 5 (2016): 749–767. 27931634 10.1016/j.bpg.2016.09.009

[22] K. Mannen , S. Tsunada , M. Hara , et al., “Risk Factors for Complications of Endoscopic Submucosal Dissection in Gastric Tumors: Analysis of 478 Lesions,” Journal of Gastroenterology 45, no. 1 (2010): 30–36, 10.1007/s00535-009-0137-4. 19760133

[23] N. G. Burgess , M. S. Bassan , D. McLeod , S. J. Williams , K. Byth , and M. J. Bourke , “Deep Mural Injury and Perforation After Colonic Endoscopic Mucosal Resection: A New Classification and Analysis of Risk Factors,” Gut 66, no. 10 (2017): 1779–1789. 27464708 10.1136/gutjnl-2015-309848

[24] M. Paspatis , J.‐M. Dumonceau , M. Barthet , et al., “Diagnosis and Management of Iatrogenic Endoscopic Perforations: European Society of Gastrointestinal Endoscopy (ESGE) Position Statement—Update 2020,” Endoscopy 52, no. 9 (2020): 792–810, 10.1055/a-1222-3191. 32781470

[25] A. Y. Wang , J. H. Hwang , A. Bhatt , and P. V. Draganov , “AGA Clinical Practice Update on Surveillance After Pathologically Curative Endoscopic Submucosal Dissection of Early Gastrointestinal Neoplasia in the United States: Commentary,” Gastroenterology 161, no. 6 (2021): 2030–2040.e1. 34689964 10.1053/j.gastro.2021.08.058

[26] World Medical Association , “Declaration of Helsinki: Ethical Principles for Medical Research Involving Human Subjects,” JAMA 310, no. 20 (2013): 2191–2194. 24141714 10.1001/jama.2013.281053

[27] Y. Saito , T. Uraoka , Y. Yamaguchi , et al., “A Prospective, Multicenter Study of 1111 Colorectal Endoscopic Submucosal Dissections (With Video),” Gastrointestinal Endoscopy 72, no. 6 (2010): 1217–1225. 21030017 10.1016/j.gie.2010.08.004

[28] H. Harada , R. Nakahara , D. Murakami , et al., “Saline‐Pocket Endoscopic Submucosal Dissection for Superficial Colorectal Neoplasms: A Randomized Controlled Trial (With Video),” Gastrointestinal Endoscopy 90, no. 2 (2019): 278–287. 30930074 10.1016/j.gie.2019.03.023

[29] N. Kobayashi , Y. Takeuchi , K. Ohata , et al., “Outcomes of Endoscopic Submucosal Dissection for Colorectal Neoplasms: Prospective, Multicenter, Cohort Trial,” Digestive Endoscopy 34, no. 5 (2022): 1042–1051. 34963034 10.1111/den.14223

[30] R. R. Singh , J. Nanavati , H. Gopakumar , and N. A. Kumta , “Colorectal Endoscopic Submucosal Dissection in the West: A Systematic Review and Meta‐Analysis,” Endoscopy International Open 11, no. 11 (2023): E1082–E1091. 38026781 10.1055/a-2181-5929 PMC10681808

[31] J. Jacques , A. Charissoux , P. Bordillon , et al., “High Proficiency of Colonic Endoscopic Submucosal Dissection in Europe Thanks to Countertraction Strategy Using a Double Clip and Rubber Band,” Endoscopy International Open 7, no. 9 (2019): E1166–E1174. 31475236 10.1055/a-0965-8531 PMC6715438

[32] M. Spychalski , M. Włodarczyk , K. Winter , J. Włodarczyk , I. Dąbrowski , and A. Dziki , “Outcomes of 601 Colorectal Endoscopic Submucosal Dissections in a Single Western Center: Is Right Colon Location Still a Major Concern?,” Surgical Laparoscopy, Endoscopy & Percutaneous Techniques 31, no. 5 (2021): 578–583, 10.1097/SLE.0000000000000940. 33935259

[33] R. de Sire , A. Capogreco , D. Massimi , et al., “Underwater Endoscopic Submucosal Dissection for Large Non‐Pedunculated Colorectal Polyps,” Gut (2025). 10.1136/gutjnl-2025-334995 40011034

[34] M. Nagata , M. Namiki , T. Fujikawa , and H. Munakata , “Prospective Randomized Trial Comparing Conventional and Underwater Endoscopic Submucosal Dissection for Superficial Colorectal Neoplasms,” Endoscopy 57, no. 5 (2025): 484–491. 39424357 10.1055/a-2445-4970 PMC12039920

[35] C. K. Oh , H. H. Chung , J. K. Park , et al., “Comparing Underwater Endoscopic Submucosal Dissection and Conventional Endoscopic Submucosal Dissection for Large Laterally Spreading Tumor: A Randomized Controlled Trial (With Video),” Gastrointestinal Endoscopy 100, no. 6 (2024): 1079–1087.e1. 38969234 10.1016/j.gie.2024.06.039

[36] H. Thorlacius , C. F. Rönnow , and E. Toth , “European Experience of Colorectal Endoscopic Submucosal Dissection: A Systematic Review of Clinical Efficacy and Safety,” Acta Oncologica 58, no. sup1 (2019): S10–S14. 30724676 10.1080/0284186X.2019.1568547

[37] A. Rimondi , G. Kalopitas , E. M. Bosch , et al., “Low Rate of General Anaesthesia and Hospital Admission Following Colonic Saline‐Immersion/Irrigation Technique (SITE) Endoscopic Submucosal Dissection (ESD),” Scandinavian Journal of Gastroenterology 60, no. 10 (2025): 932–937. 40677121 10.1080/00365521.2025.2531436

[38] M. Nagata , “Usefulness of Underwater Endoscopic Submucosal Dissection in Saline Solution With a Monopolar Knife for Colorectal Tumors (With Videos),” Gastrointestinal Endoscopy 87, no. 5 (2018): 1345–1353, https://www.sciencedirect.com/science/article/pii/S0016510717325403. 29242059 10.1016/j.gie.2017.11.032

[39] Y. Koyama , M. Fukuzawa , H. Aikawa , et al., “Underwater Endoscopic Submucosal Dissection for Colorectal Tumors Decreases the Incidence of Post‐Electrocoagulation Syndrome,” Journal of Gastroenterology and Hepatology 38, no. 9 (2023): 1566–1575. 37321649 10.1111/jgh.16259

[40] S. Singh , B. P. Mohan , R. Vinayek , et al., “Underwater Versus Conventional Endoscopic Submucosal Dissection for Colorectal Lesions: Systematic Review and Meta‐Analysis,” Gastrointestinal Endoscopy 101, no. 3 (2025): 551–557.e5, 10.1016/j.gie.2024.10.029. 39427993

